# AKT activation controls cell survival in response to HDAC6 inhibition

**DOI:** 10.1038/cddis.2016.180

**Published:** 2016-06-30

**Authors:** M Kaliszczak, S Trousil, T Ali, E O Aboagye

**Affiliations:** 1 Cancer Imaging Centre, Department of Surgery and Cancer, Imperial College London, Hammersmith Hospital, Du Cane Road, London W12 0NN, UK

## Abstract

HDAC6 is emerging as an important therapeutic target for cancer. We investigated mechanisms responsible for survival of tumor cells treated with a HDAC6 inhibitor. Expression of the 20 000 genes examined did not change following HDAC6 treatment *in vivo*. We found that HDAC6 inhibition led to an increase of AKT activation (P-AKT) *in vitro*, and genetic knockdown of HDAC6 phenocopied drug-induced AKT activation. The activation of AKT was not observed in PTEN null cells; otherwise, *PTEN/PIK3CA* expression *per se* did not predict HDAC6 inhibitor sensitivity. Interestingly, HDAC6 inhibitor treatment led to inactivating phosphorylation of PTEN (P-PTEN Ser380), which likely led to the increased P-AKT in cells that express PTEN. Synergy was observed with phosphatidylinositol 3'-kinases (PI3K) inhibitor treatment *in vitro,* accompanied by increased caspase 3/7 activity. Furthermore, combination of HDAC6 inhibitor with a PI3K inhibitor caused substantial tumor growth inhibition *in vivo* compared with either treatment alone, also detectable by Ki-67 immunostaining and ^18^F-FLT positron emission tomography (PET). In aggregate AKT activation appears to be a key survival mechanism for HDAC6 inhibitor treatment. Our findings indicate that dual inhibition of HDAC6 and P-AKT may be necessary to substantially inhibit growth of solid tumors.

The acetylation status of protein lysines including that of histones is regulated by the reversible post-translational modification activities of histone deacetylases (HDACs; more accurately, lysine deacetylates) and histone acetyltransferases. Because these proteins are deregulated in cancer, there is a strong interest to inhibit their function. HDACs fall into four classes consisting of 18 genes,^1^ including zinc-dependent class I (HDACs 1, 2, 3 and 8), II (HDACs 4, 5, 6, 7, 9 and 10) and IV (HDAC 11) enzymes, and nicotinamide adenine dinucleotide-dependent class III enzymes (sirtuins). Although most clinically relevant HDAC inhibitors developed to date represent drugs that modify chromatin – the prototype epigenetic therapy – compounds that target the class IIb HDAC, HDAC6 are distinguished by their ability to deacetylate non-histone substrates. HDAC6 inhibition has recently emerged as an attractive target for the treatment of cancer. HDAC6 was shown to deacetylase a diverse set of substrates involved in tumorigenesis including HSP90, α-tubulin, cortactin and peroxiredoxins, but, importantly, unlike other histone deacetylases, selective inhibition of HDAC6 is believed not be associated with severe toxicity and HDAC6 knockout does not lead to embryonic lethality.^2, 3, 4, 5, 6^ The role of HDAC6 in the misfolded/damaged proteins response, particularly important for tumor cells that produce large amounts of these aberrant proteins has also been exploited.^7^ A HDAC inhibitor with enhanced selectivity for HDAC6, ACY-1215, is currently being tested in phase I/II against refractory multiple myeloma in combination with proteasome inhibitor bortezomib (clinical trial NCT 01323751). HDAC6 inhibitors have been less studied in the context of solid tumors.

Phosphatidylinositol 3'-kinases (PI3K) are lipid kinases that catalyze production of phosphatidylinositol 3,4,5-triphosphate, which in turn functions to recruit and activate several cognate targets including AKT. PI3K activation gain of function can occur through amplification or mutation of *PIK3CA* located on chromosome 3q26.3 that encodes PI3K p110*α*. Loss of function can occur through mutation of *PTEN*, a tumor suppressor gene on chromosome 10q23, which encodes a dual-specificity lipid and protein phosphatase that negatively regulates AKT. Co-existence of both mutations has also been reported in endometrial cancer.^8^ PTEN inactivation leads to AKT phosphorylation and consequently activation of multiple downstream substrates including the mTOR proteins, caspases, cell cycle proteins and NF-*κ*B to promote cell survival, metastasis and chemoresistance.^9, 10^ Activation of the PI3 kinase-AKT pathway represents one of the major mechanisms employed by cancer cells for cell survival, and by extension the pathway is often deregulated in cancer cells that are refractory to chemotherapy. For example DNA-PK-dependent AKT activation is implicated in acquired platinum-resistant ovarian cancer cells,^11^ whereas PTEN-dependent AKT activation is associated with resistance to small molecule EGFR kinase inhibitors in lung cancer.^12^

We previously described a novel HDAC6 selective inhibitor, C1A, that showed antitumor activity in a solid tumor model of human colorectal cancer.^13^ The molecular mechanisms by which tumors survive HDAC6 inhibitor treatment, however, remain unresolved. Here we investigated potential survival mechanism using C1A as a model HDAC6 inhibitor active in solid tumors. Our results reveal a previously unknown mechanism by which AKT activation regulates survival following HDAC6 inhibitor treatment.

## Results

### Mechanisms underlying loss of growth response during HDAC6 inhibition

To understand potential drug resistance genetic mechanisms, we performed a gene array analysis on tumor samples (HCT-116 colon cancer xenografts: a subcutaneously growing tumor derived from injecting the HCT-116 cell line into mice) obtained from mice treated daily with C1A for 14 days – a time point when recovery from drug inhibition was seen.^13^ In total, 20000 genes were tested (GEO accession number: GSE64662; http://www.ncbi.nlm.nih.gov/geo/query/acc.cgi?acc=GSE64662). A representative set of the genes is shown in Table 1. After adjustment for multiple testing using Benjamini–Hochberg, none of the genes was significantly expressed between the control and treated groups at either the *q*<0.05 or *q*<0.1 levels. Furthermore, no obvious genotypes linked to a classical resistance mechanism (e.g., de-regulation of the ABC transporters) were found. Furthermore, C1A was found to be a weak substrate of the ABC transporters, ABCG2, P-gp and MRP1 (Supplementary Figure S1).

**Figure 1:**
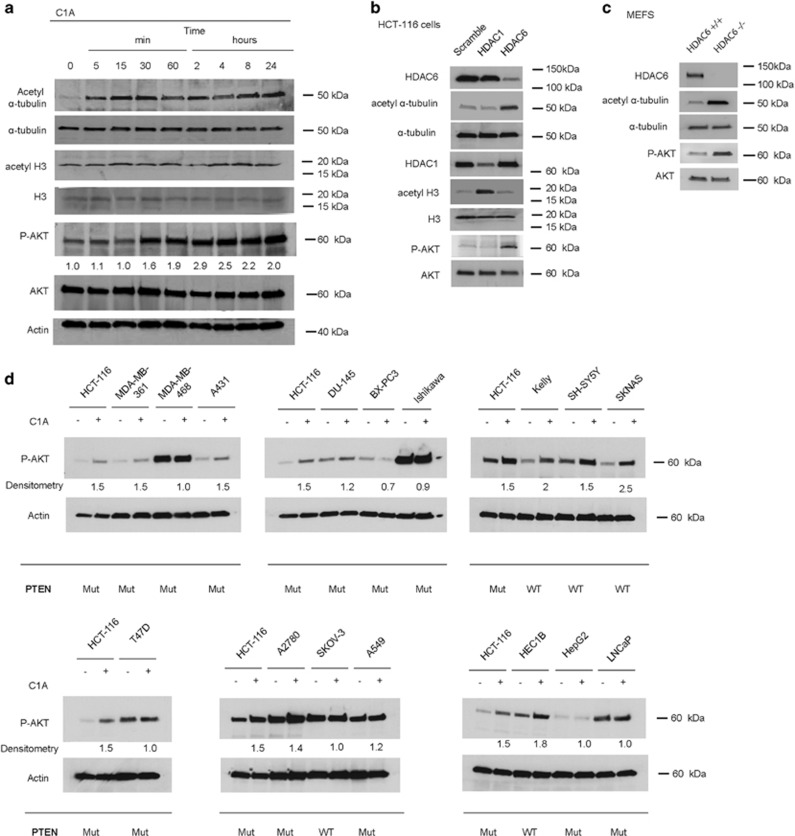
HDAC6 inhibition induces AKT phosphorylation. (**a**) P-AKT levels following treatment with C1A at 10 *μ*M for the indicated time in HCT-116 cells. Corresponding data from same lysates for acetyl *α*-tubulin, *α*-tubulin, acetyl histone H3 (acetyl H3), H3, total AKT (AKT) and *β*-actin (actin) are shown. Densitometry data are expressed as fold increase over control (P-AKT is normalized to AKT). (**b**) Impact of genetic knockdown of HDAC1 and HDAC6 by siRNA on their respective substrates, acetyl H3 and acetyl *α*-tubulin, and on P-AKT levels in HCT-116 cells. (**c**) P-AKT expression in Mouse Embryonic Fibroblasts (MEF) HDAC6 WT and HDAC6^−/−^, relative to expression of HDAC6 and acetyl *α*-tubulin. (**d**) Effect of HDAC6 inhibition by C1A on P-AKT levels in a panel of cancer cell lines. Cells were treated with 10 *μ*M of C1A for 8 h and subject to western blotting. Densitometry data are expressed as fold increase over control (P-AKT is normalized to AKT). Mutation status of PTEN is indicated: mutant (Mut) or wildtype (WT). The human cell lines used and their background include: HCT-116 (colon carcinoma; primary), MDA-MB-361 (breast adenocarcinoma; brain metastasis), MDA-MB-468 (breast adenocarcinoma; plural effusion metastasis), A431 (skin carcinoma; primary), DU-145 (prostate carcinoma; brain metastasis), BX-PC3 (pancreatic adenocarcinoma; primary), Ishikawa (endometrial adenocarcinoma; primary), Kelly (neuroblastoma; brain), SH-SY5Y (neuroblastoma and subline of parental line SK-N-SH; bone marrow metastasis), SKNAS (neuroblastoma; bone marrow), T47D (breast adenocarcinoma; plural effusion metastasis), A2780 (ovarian carcinoma; primary), SKOV-3 (ovarian adenocarcinoma; ascites), A549 (lung adenocarcinomic; primary), HEC1B (endometrial adenocarcinoma; primary), HepG2 (hepatocellular carcinoma; primary) and LNCaP (prostate carcinoma; supraclavicular lymph node metastasis)

**Figure 2:**
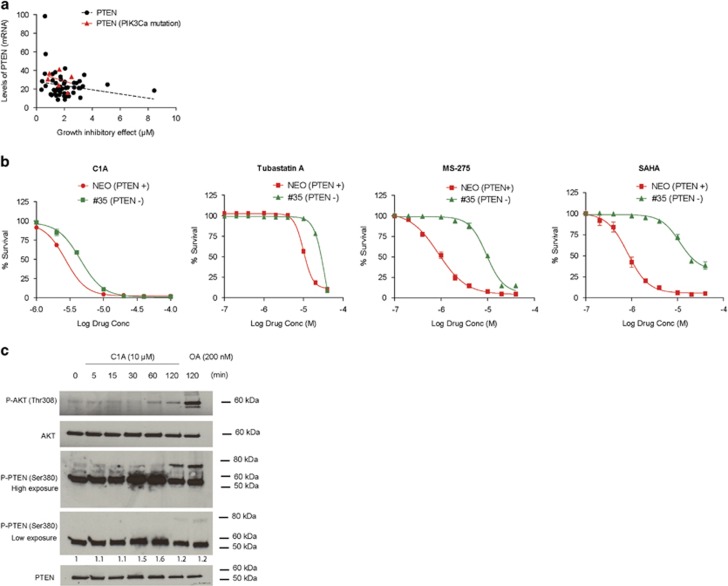
HDAC6 inhibition in relation to PTEN expression and activation. (**a**) Relationship between the growth inhibitory effect of C1A and mRNA levels of PTEN. The growth inhibitory effect of C1A was tested across the NCI60 panel following 72 h exposure. Data on mRNA levels and PIK3CA mutation status were from the Developmental Therapeutics Program (NCI/NIH) available online. (**b**) Growth inhibitory effect of HDAC inhibitors in HCT-116 cells proficient (NEO) and deficient (#35) in PTEN.^18^ HCT-116 Neo and #35 were treated with HDAC6 inhibitors (C1A and Tubastatin A), pan HDAC inhibitor (SAHA) and HDAC class I-specific inhibitor (MS-275) for 24 h. All compounds were used at 10 *μ*M. (**c**) Impact of C1A treatment on PTEN phosphorylation. P-PTEN (Ser380) expression was compared to total PTEN, P-AKT (Thr308) and total AKT expression. Densitometry data are the ratios between P-PTEN and PTEN and normalized to untreated control. For clarity, two exposure times for western blot were used

AKT represents one of the most important proteins that is adaptively regulated in relation to cancer cell survival, and increased phospho-AKT (P-AKT) is thought to contribute to an apoptotic resistance phenotype.^14, 15, 16^ HDAC6 inhibition by C1A (increased acetyl *α*-tubulin with no change in acetyl H3) was found to be associated with a time-dependent increase of P-AKT (Figure 1a). Genetic knockdown of HDAC6 in HCT-116 or mouse embryonic fibroblast (MEF) cells phenocopied treatment with C1A in relation to P-AKT accumulation (Figures 1b and c). Treatment-induced increased P-AKT also occurred in a panel of cancer lines from different origins and *PTEN* mutation status^8, 17^ (Figure 1d). For each set of cell lines, HCT-116 cell lysates (heterozygous for *PIK3CA*, c.3140A>G, and homozygous for *PTEN*, c.68_69insG) were used to permit optimal display of protein expression. Increased P-AKT was seen in both *PTEN* mutant and wild-type cell lines inferring that mutation status *per se* did not explain the increased P-AKT. HEC1B cells that are wildtype for *PTEN* but harbor mutant *PIK3CA* and *KRAS*^8^ also showed increased P-AKT activity. PTEN protein loss can occur through promoter methylation, loss of heterozygosity and regulation at the RNA or protein level making examination of mutational status alone insufficient to predict protein function. P-AKT expression levels were very high in two cell lines (MDA-MB-468 and Ishikawa) that are null for PTEN protein,^8, 17^ relative to that in HCT-116 cells, and importantly showed no change following C1A treatment (Figure 1d), suggesting that PTEN protein may have a role in the activation of AKT following treatment with a HDAC6 inhibitor. To rule out the notion that PTEN expression *per se* may predict cell line sensitivity to C1A, we evaluated the association between C1A-dependent growth inhibition of the NCI60 cell line panel and expression of *PTEN* mRNA, and observed no linear association between growth and *PTEN* expression levels (Figure 2a). In isogenic HCT-116 and HCT-116 PTEN null cells, cell survival following HDAC6 inhibitor treatment with C1A or tubastatin A was marginally higher in the PTEN null cells (Figure 2b); in contrast PTEN null cells were substantially more resistant to treatment with MS-275 (Class I HDAC inhibitor) or SAHA (a pan HDAC inhibitor), indicating differences in drug–response profile.^18^

**Figure 3:**
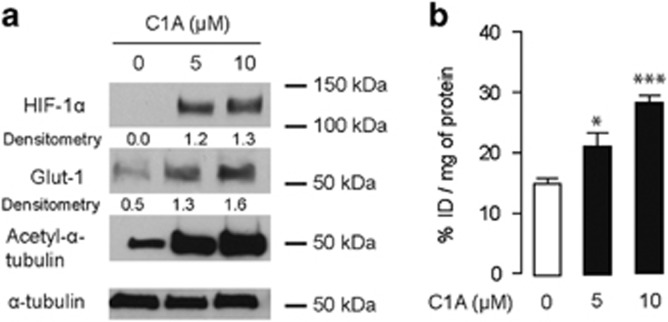
HDAC6 inhibition induces HIF1*α*-mediated response, Glut1 expression and glucose uptake. (**a**) HCT-116 cells were treated with C1A at 5 *μ*M and 10 *μ*M for 4 h and subject to western blotting. Densitometry data were normalized to tubulin levels. (**b**) [^18^F]FDG uptake in HCT-116 cells following treatment with C1A at 5 and 10 *μ*M for 4 h (expressed as a % of the injected dose per mg of cellular protein). *P=0.036, ***P<0.0001

We wondered if PTEN activity rather than expression may be responsible for the HDAC6 inhibitor-induced AKT activation. We investigated phosphorylation of the PTEN C-terminal serine–threonine cluster.^19, 20^ Treatment with C1A increased phospho-PTEN (P-PTEN Ser380) expression at 30–60 min (Figure 2c). A higher molecular weight band was observed at 120 min, possibly owing to additional post-translational modifications of PTEN, similar to that observed with Okadaic acid (Figure 2c). We postulated that C1A treatment decreases PTEN lipid phosphatase activity via phosphorylation and consequently activates P-AKT (P-AKT-Th308; Figure 2c). We further investigated whether C1A treatment also activated AKT downstream substrates: hypoxia-inducible factor-1 *α* and glucose trasporter-1 (GLUT1).^21^ Both HIF1-α and GLUT1 protein expression increased upon 4 h of C1A treatment at 5 or 10 *μ*M (Figure 3a). Uptake of ^18^F-fluorodeoxyglucose ([^18^F]FDG) also increased with C1A treatment at 10 *μ*M C1A by twofold in keeping with the higher GLUT1 protein expression (Figure 3b). This demonstrates functional significance of the drug-induced increased P-AKT.

### Combination therapy with AKT pathway inhibitors

**Figure 4:**
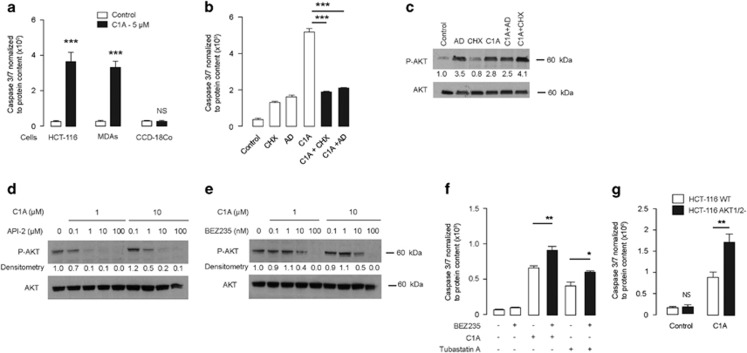
HDAC6 inhibition induces caspase 3/7 activation that is potentiated by PI3K/AKT inhibition. (**a**) Caspase 3/7 activity following 24 h treatment with vehicle (control) or C1A at 5 *μ*M in HCT-116, MDA-MB-231 and CCD-18Co cells. Data were normalized to protein content (***P<0.0001). (**b**) Impact of transcription inhibitor actinomycin D (AD – 10 *μ*g/ml) and protein synthesis inhibitor cycloheximide (CHX – 5 *μ*g/ml) on caspase 3/7 activity following treatment with HDAC6 inhibitors (24 h treatment) (***P<0.0001). (**c**) Impact of actinomycin D and cycloheximide as above on P-AKT and total AKT expression. (**d** and **e**) Effect of API-2 and BEZ235 on AKT activation mediated by C1A. The compounds were co-incubated for 24 h at the indicated concentrations. (**f**) Effect BEZ235 (100 nM) on caspase 3/7 activation mediated by C1A (10 *μ*M) or tubastatin A (10 *μ*M). The compounds were co-incubated for 24 h as indicated **P=0.0055, *P=0.0205. (**g**) Effect of C1A on wild-type HCT-116 (WT) and HCT-116 cells with knockout of AKT1/2 (AKT1/2-). Cells were treated for 6 h at 40 *μ*M, washed and left for further 18 h. **P=0.0051

Using caspase 3/7 activity as a surrogate for apoptotic cell death induction, we showed that C1A treatment-induced apoptosis in both HCT-116 human colon and MDA-MB-231 human breast cell lines by 3.7- and 3.5-fold, respectively, but not in CDC-18Co normal colon fibroblast cell line (Figure 4a). To gain some insight into the molecular mechanisms, we examined the effect of the transcription inhibitor actinomycin D and the translation inhibitor cycloheximide. Both actinomycin D and cycloheximide abrogated caspase 3/7 activation induced by C1A (Figure 4b). These data suggest that *de novo* synthesis of pro-apoptotic factors or repression of anti-apoptotic factors accompanies apoptosis induced by C1A treatment. While we did not investigate the specific factors involved, two pro-apoptotic genes – BAX and XAF1 – were previously reported by us to be upregulated *in vivo* following C1A treatment.^13^ Surprisingly, neither actinomycin D nor cycloheximide prevented the HDAC6 inhibitor-induced increase of P-AKT by C1A (Figure 4c), suggesting that the two process resulting from C1A treatment – apoptosis induction and AKT activation – are mechanistically distinct.

From the foregoing, we rationalized that a therapeutic combination strategy would involve HDAC6 inhibition together with inhibition of AKT phosphorylation. Simultaneous exposure of HCT-116 cells to C1A and API-2 (pan-AKT inhibitor) or BEZ235 (dual PI3K/mTOR inhibitor) retarded the C1A induction of P-AKT expression (Figure 4d). Increased caspase 3/7 activity was observed when BEZ235 was combined with HDAC6 inhibitors C1A or tubastatin A (Figure 4e). To provide a genetic basis for the above findings, we further treated HCT-116 or isogenic AKT1/2 knockout cells with C1A. Caspase 3/7 activity was higher in the latter cell line (Figure 4f).^22^ Regarding efficacy, synergy was seen when HDAC6 inhibitor treatment was combined with a variety of PI3K/AKT/mTOR inhibitors including rapamycin, wortmanin, LY29004, BEZ235 and API-2 (Supplementary Table S1).

**Figure 5:**
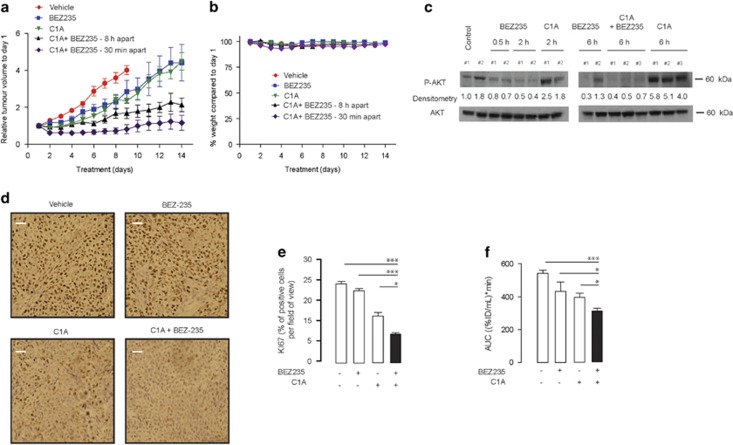
AKT inhibition potentiates the activity of HDAC6 inhibitor treatment *in vivo*. (**a**) Nude mice bearing HCT-116 tumor xenograft were administered BEZ235 at 25 mg/kg QD. (p.o.) or C1A at 20 mg/kg (QD) (i.p.) alone or in combination (30 min or 6 h apart). Relative tumor volumes±s.e.m. are shown. *n*=8 per group. (**b**) Body weight measurements following treatment as measure of systemic drug tolerability. (**c**) P-AKT expression measured in HCT-116 tumors treated with BEZ235 (25 mg/kg), C1A (20 mg/kg) or combination (no delay between injections) followed by excision of the tumor (at the indicated time post treatment) and analysis by western blotting. Densitometry data of P-AKT were normalized to AKT and to vehicle-treated controls. (**d**) Representative images of Ki-67 immunostained tumor sections showing proliferative Ki-67 positive cells (*brown*). Scale bars, 10 *μ*m. (**e**) Summary of Ki-67 staining from 10 random pictures per slice (*n*=2 slices per tumor per group); the positive cells were counted using Image J software. The Ki-67 positive cells were expressed as a percentage of total cells counted. All error bars show s.e.m. (*** *P*<0.0001, two tailed student *t*-test). (**f**) [^18^F]FLT uptake following treatment for 2 days with BEZ235 or/and in combination with C1A. Mice were treated with BEZ235 (at 25 mg/ kg QD) or with C1A (at 20 mg/kg QD) alone or in combination (30 min apart) *n*=3. The area under the curve represent the [^18^F]FLT uptake over 60 min

To verify whether AKT inhibition potentiated the efficacy of a HDAC6 inhibitor *in vivo*, HCT-116 tumor-bearing mice were treated with C1A in combination with BEZ235. C1A treatment alone was associated with a Tumor Growth Delay (TGD_2x_) of 3.8±1.3 days and a Total Growth Inhibition (TGI) of 69% (Figure 5a). Combination treatment (given 6 h apart) was associated with a TGD_2x_ of 8.2±1.3 days and a TGI of 74%, and the effect was more pronounced when drugs were given 30 min apart (TGI of 115% TGD_2x_ cannot be calculated in this case). No toxicity as measured by body weight loss was observed (Figure 5b).

These data indicate that, when combined appropriately, a drug that inhibits P**-**AKT can positivily modulate the activity of a HDAC6 inhibitor as demonstrated with C1A. P-AKT expression was low at 30 min following injection of BEZ235 (Figure 5c). Comparatively, single treatment of C1A showed higher P-AKT expression that was retarded by the combination regimen at 6 h. Efficacy of the combination treatment in tumors could be predicted by immunostaining the proliferative biomarker Ki-67 in excised tumors obtained at 48 h or by non-invasive imaging with the proliferation marker [^18^F]fluorothymidine ([^18^F]FLT)-PET^23^ at 48 h (Figures 5d and e).

## Discussion

HDAC6 is emerging as an important therapeutic target for cancer. We investigated mechanisms responsible for survival of tumor cells treated with a HDAC6 inhibitor and report that HDAC6 inhibition promotes inactivating PTEN phosphorylation and consequently activation of AKT. In the development of new drugs, it is important to ascertain mechanisms of resistance so as to optimize treatment outcome. Previous studies documented that the HDAC6 inhibitor C1A induced apoptosis in cell lines derived from solid tumors, however, tumor growth survival occurred in spite of continued drug treatment in mice.^13^ Our studies subsequently unraveled a mechanism of resistance involving inactivating PTEN phosphorylation.^24^

Other than gene expression, the lipid phosphatase activity and protein stability of PTEN can be regulated through acetylation, phosphorylation or ubiquitination.^25^ HDAC6 inhibition was recently reported to induce PTEN acetylation and activity;^26^ however, we found no differences in acetylated-lysine of PTEN by mass spectrometry following C1A treatment of HCT-116 cells (data not shown). We also considered activation of *PIK5-L1* gene that is enhanced at an early time point (24 h) following C1A treatment as a potential reason for increased P-AKT,^13, 27^ however, we ruled out this possibility given the persistence of P-AKT increase when transcription or translation was blocked. PTEN is also subject to phosphorylation at the C-terminal serine–threonine cluster (Ser370, Ser380, Thr382, Thr383 and Ser385) that affects its phosphatase activity by restricting the protein to the cytoplasm and away from the plasma membrane where it antagonizes PI3K/AKT signaling.^19, 20^ Treatment with C1A increased phospho-PTEN (P-PTEN Ser380) expression at 30–60 min. The higher molecular weight band observed at 120 min is possibly due to additional post-translational modifications of PTEN, similar to that observed with Okadaic acid in our study and that of others.^24^ We postulate that C1A treatment decreases PTEN lipid phosphatase activity via phosphorylation and consequently activates PI3K-AKT.

Two opposing processes – caspase 3/7 activation and AKT-dependent survival – occurred as a consequence of HDAC6 inhibition. We report that the mechanisms are distinct; cell death was dependent on transcription and translation, whereas AKT activation was not. Treatment of tumor cells or xenografts with PI3K/AKT/mTOR pathway inhibitors abrogated the increased P-AKT expression and enhanced antitumor activity of C1A at well-tolerated doses. The effect was schedule dependent. It could be argued that inhibition of the PI3K/AKT/mTOR axis will also allow HDAC6 inhibition to be more effective in cells that already harbor inactivating mutations or deletions of PTEN (hence, constitutive P-AKT), even though these cells do not respond to HDAC6 inhibition by activating P-AKT.

Regarding mechanistic biomarkers of efficacy, the combination of C1A and BEZ235 could be monitored with [^18^F]FLT-PET in HCT**-**116 tumor-bearing mice as early as 48 h. Interestingly, our result also indicate that, following activation of AKT pathway and GLUT1 expression, HCT-116 cells upregulate glucose uptake, demonstrated as an increase of [^18^F]FDG-PET uptake at 4 h after treatment. PET imaging could therefore be used in the pre-clinical and clinical settings to monitor both anti-proliferative and survival mechanism following treatment with HDAC6 inhibitors.

In conclusion, inactivating PTEN phosphorylation and AKT activation appear to be key survival mechanisms for HDAC6 inhibitor treatment of solid tumors. Our findings indicate that dual inhibition of HDAC6 and P-AKT may be necessary to substantially inhibit growth of solid tumors.

## Materials and Methods

### Compounds

C1A was synthesized in house,^13^ SAHA and tubastatin A were purchased from Cayman Chemical (Ann Arbor, MI, USA). API-2 was from obtained from Tocris (Abingdon, UK). BEZ235 was from LC Laboratories (Woburn, MA, USA). Cycloheximide, actinomycin D and LY29004 were from Calbiochem (San Diego, CA, USA). Okadaic acid was purchased from Cell Signalling (Danvers, MA, USA). Fumitremorgin C, MK-571, vinblastine, rapamycin, wortmanin and LY29004 were from Sigma (St. Louis, MO, USA).

### Antibodies

Antibodies against P-AKT (Ser473, catalog 9271), P-AKT (Thr308, catalog 9275) and AKT (catalog 9272), HDAC1 (catalog 5356), HDAC6 (catalog 7558), P-PTEN (Ser380, catalog 9551) and PTEN (catalog 9188), *α*-tubulin (catalog 2144), acetyl *α*-tubulin (catalog 5335), Histone H3 (catalog 9715), P-DNA-PK (Ser2056, catalog 4215), acetyl lysine (catalog 9441) and HIF1-*α* (catalog 3716) were from Cell Signalling. Antibody against acetyl histone H3 (catalog 06-599) was from Millipore (Nottingham, UK). Glut1 (catalog ab40084) and β-actin (catalog ab6276) antibodies were from Abcam (Cambridge, UK). Secondary goat anti-mouse (catalog sc-2005) and anti-rabbit (catalog sc-2004) HRP antibodies were from Santa Cruz (Heidelberg, Germany).

### Cells

HCT-116 cells were obtained from the American Type Cell Culture Collection and authenticated by short tandem repeat profiling under contract by DDC Medical (London, UK). Ishikawa was purchased from Sigma. MEFs proficient (WT) and deficient (KO) in HDAC6 were kindly provided by Professor Tso-Pang Yao (Duke University, USA); KELLY, SH-SY5Y and SKNAS by Dr Louis Chesler (Institute of Cancer Research, UK), AKT1/AKT2 HCT-116-deficient cells from Professor Bert Vogelstein (Johns Hopkins University, USA), PTEN knockout variants of HCT-116 cells were generously provided by Dr Todd Waldman (Georgetown University, USA). 3T3 and 3T3 transfected with c-DNA expressing P-gp (pHamdr1) were kindly provided by Dr. E Schuetz from St. Jude's Children Research Hospital (USA) and MCF7 and mitoxantrone (MX)-resistant sub-clones MCF7-MX by Dr E Schneider from the University of Maryland (USA). All other cell lines were obtained from ATCC. All cells were passaged in our laboratory for fewer than 6 months on receipt and were tested mycoplasma free.

### Growth inhibitory assay

Drug concentrations that inhibited 50% of cell growth (GI_50_) were determined using a sulforhodamine B (SRB) technique as described elsewhere.^28^

### Tumor xenografts

All animal experiments were done by licensed investigators in accordance with the United Kingdom Home Office Guidance on the Operation of the Animal (Scientific Procedures) Act 1986 (HMSO, London, UK, 1990) and within guidelines set out by the UK National Cancer Research Institute Committee on Welfare of Animals in Cancer Research.^29^ HCT-116 (5 x 10^6^) cells were injected subcutaneously in 100 *μ*l volumes into the flank of female nu/nu-BALB/c mice (Harlan, Blackthorn, UK). Animals entered treatment groups when tumors reached ~50 mm^3^. Animal weights and tumor volumes were determined daily.

### Gene array

Animals were treated for 14 days with C1A at 40 mg/kg^/^day (or vehicle) and the tumors excised and snap frozen. Total RNA was extracted from tumors using RNeasy mini Kit (Qiagen, Manchester, UK) and hybridized to Affymetrix human genome U219 microarray (Affymetrix, Santa Clara, CA, USA). Studies were performed under contract by AlphaMetrix biotech (Rödermark, Germany). Raw data were normalized using robust multi-chip average in R.^30^ Differential gene expression analysis was conducted using the ‘limma' package and the adjusted *P*- value was derived by the Benjamini & Hochberg method.^31^

### Immunoblotting

Cells and tumor tissue samples were prepared and subject to western blotting as previously described.^32^ Densitometry ratios were determined using Image J software (Rasband, W.S., Image J, US National Institutes of Health, Bethesda, MD, USA, http://imagej.nih.gov/ij/, 1997-2015).

### *In vitro* [^18^F]FDG-cell uptake

Cells were seeded on day 1 and on day 2, fresh growth medium, containing ~0.74MBq [^18^F]FDG, was added to individual wells. Cell uptake was measured following incubation at 37 °C in a humidified atmosphere of 5% CO_2_ for 1 h. The plates were subsequently placed on ice, washed with ice-cold PBS and lyzed in RIPA buffer. Cell lysates were transferred into counting tubes and decay-corrected radioactivity was determined on a gamma counter (Cobra II, Packard, PerkinElmer, Beaconsfield, UK). Data were expressed as percentage of total radioactivity incorporated into cells, normalized for total cellular protein.

### Caspase 3/7 assay

Caspase 3/7 activity was determined using Promega's caspase 3/7 assay according to the manufacturer's instructions. In brief, cells were transferred to a white opaque 96-well plate, incubated for 1 h with Caspase-Glo reagent and the enzymatic activity of caspase 3/7 was measured using a TopCount counter and normalized with the protein content.

### SiRNA knockdown

Transient knockdown of HDAC1, HDAC6 in HCT-116 cells were performed using ON-TARGETplus SMARTpool siRNAs (Dharmacon, Lafayette, CO, USA) with wet reverse transfection according to manufacturer's instructions. RNAiMAX (Invitrogen) was used as transfection reagent.

### [^18^F]FLT tumor PET imaging

HCT-116 (5 × 10^6^) cells were injected on the back of female nu/nu-BALB/c athymic nude mice. When xenografts reached ~100 mm^3^, HCT-116 tumor-bearing mice were treated with C1A or BEZ235 alone or in combination. At 48 h post treatment, the animals were scanned on a dedicated small animal PET scanner (Siemens Inveon PET module; Siemens, Erlangen, Germany) following a bolus intravenous injection of 3.7 MBq of [^18^F]fluorothymidine ([^18^F]FLT) and tumor uptake analyzed as previously described.^32^ Dynamic emission scans were acquired in list-mode format over 60 min. The acquired data were then sorted into 0.5 mm sinogram bins and 19 time frames for image reconstruction, which was done by filtered back projection. Cumulative images of the data (30–60 min) were used for visualization of radiotracer uptake and to define the regions of interest (ROIs) across several slices with the Siemens Inveon Research Workplace software (3D ROIs were defined for each tumor). The count densities were averaged for all ROIs at each of the 19 time points to obtain a time versus radioactivity curve. The radiotracer uptake in tumor regions was normalized to that of the heart of the same animal. The normalized uptake value at 60 min post injection (NUV60) was used for comparisons. The area under the NUV curve was calculated as the integral of NUV from 0 to 60 min.

### Ki-67 immunostaining

For histological evaluation of the degree of tumor proliferation, tumors were excised after imaging, fixed in formalin, embedded in paraffin and cut into 5.0 *μ*m sections and subject to Ki-67 immunostaining and analysis as previously described.^32^

### Combination studies *in vitro*

HCT-116 cells were seeded on day 1 in a volume of 100 *μ*l and treated on day 2 with a range of concentration of C1A and tubastatin A around the GI_50_ (0.1–40 *μ*M) in a volume of 50 *μ*l. Simultaneously, different inhibitors were added at the indicated concentration in a volume of 50 *μ*l. The cells were incubated for an additional 72 h and the growth inhibitory effect was evaluated using the SRB assay. The combination index was determined using the CalcuSyn software version 2.1. CI<1 indicates synergistic effect; CI=1, additive effect; and CI>1, no significant combination effect.

### Permeability

Permeability of test compounds was performed using the transwell assay as previously described.^33^

### Statistical analysis

Two tailed student's *t*-tests were performed using GraphPad prism software, and *P-*values <0.05 using a 95% confidence interval were considered significant. Data are reported as mean±S.E.M. of at least three independent experiments unless otherwise stated. *P<0.05, **P<0.005, ***P<0.0001. NS, not significant.

## Figures and Tables

**Table 1:** Differential gene expression analysis of HCT-116 tumors following 14 days of treatment with C1A at 40 mg/kg/day compared with vehicle-treated tumors (*n*=3 per group)

**Gene symbol**	**logFC**	***P*-value**	**Adjusted** ***P*-value**	**Gene title**
NTHL1	−0.736677656	2.55E-05	0.777442325	Nth endonuclease III-like 1 (*E. coli*)
ALCAM	−0.920675312	0.000112974	0.777442325	Activated leukocyte cell adhesion molecule
ZNF655	0.766978363	0.000116352	0.777442325	Zinc finger protein 655
URB2	0.865943736	0.000119016	0.777442325	URB2 ribosome biogenesis 2 homolog (*S. cerevisiae*)
NR3C1	−0.799750027	0.000119338	0.777442325	Nuclear receptor subfamily 3, group C, member 1 (glucocorticoid receptor)
FLYWCH1	0.679778646	0.000179763	0.777442325	FLYWCH-type zinc finger 1
CSGALNACT1	−0.882311251	0.000199763	0.777442325	Chondroitin sulfate *N-*acetylgalactosaminyltransferase 1
PPT2	0.711507486	0.000227027	0.777442325	Palmitoyl-protein thioesterase 2
ALDH1B1	0.753485489	0.000240906	0.777442325	Aldehyde dehydrogenase 1 family, member B1
ALDH8A1	0.712958175	0.000293548	0.777442325	Aldehyde dehydrogenase 8 family, member A1
RPS27A	0.686609012	0.000311095	0.777442325	Ribosomal protein S27a
C6orf57	−0.579207261	0.000320163	0.777442325	Chromosome 6 open reading frame 57
SAMD5	0.736334559	0.000337219	0.777442325	Sterile alpha motif domain-containing 5
ALDH1B1	0.678048588	0.00033818	0.777442325	Aldehyde dehydrogenase 1 family, member B1
MYBBP1A	0.563704418	0.000340876	0.777442325	MYB binding protein (P160) 1a
KLK3	−0.606943925	0.000422135	0.777442325	Kallikrein-related peptidase 3
GFOD1	0.617851796	0.000447255	0.777442325	Glucose-fructose oxidoreductase domain-containing 1
SMUG1	0.583214452	0.000477869	0.777442325	Single-strand-selective monofunctional uracil-DNA glycosylase 1
SAPS2	0.871468969	0.00048278	0.777442325	SAPS domain family, member 2
SEMA3C	−0.924371191	0.000506638	0.777442325	Sema domain, immunoglobulin domain (Ig), short basic domain, secreted, (semaphorin) 3C
TMCC3	−0.600920858	0.000523683	0.777442325	Transmembrane and coiled-coil domain family 3
B4GALT2	0.513925699	0.000532725	0.777442325	UDP-Gal:betaGlcNAc beta 1,4- galactosyltransferase, polypeptide 2
CPEB1	−0.556732661	0.000571699	0.777442325	Cytoplasmic polyadenylation element binding protein 1
AKR1C3	−0.649604098	0.000591514	0.777442325	Aldo-keto reductase family 1, member C3 (3-alpha hydroxysteroid dehydrogenase, type II)
ITGB8	−0.847908487	0.000593268	0.777442325	Integrin, beta 8
LOC441155/ZC3H11 A	−0.710048175	0.000617314	0.777442325	Zinc finger CCCH-type domain-containing pseudogene/zinc finger CCCH-type containing 11A
BDH1	0.57030334	0.000617955	0.777442325	3-hydroxybutyrate dehydrogenase, type 1
SLC43A3	0.674730913	0.000653629	0.777442325	Solute carrier family 43, member 3
RUNX1	−0.623212555	0.000703953	0.777442325	Runt-related transcription factor-1
EIF4A1	0.527807794	0.000712078	0.777442325	Eukaryotic translation initiation factor 4A1
FOXD4/FOXD4L1	0.574366602	0.000724742	0.777442325	Forkhead box D4/forkhead box D4-like 1
SERPINF1	−0.873740093	0.000726528	0.777442325	Serpin peptidase inhibitor, clade F (alpha-2 antiplasmin, pigment epithelium derived factor), member 1
SCAMP5	0.491463362	0.000734319	0.777442325	Secretory carrier membrane protein 5
PAK6	0.584001518	0.000749791	0.777442325	p21 protein (Cdc42/Rac)-activated kinase 6

## Abbreviations

HDAC: histone deacetylase
PTEN: phosphatase and tensin homolog
PIK3CA: phosphatidylinositol-4,5-bisphosphate 3-kinase catalytic subunit alpha
^18^F-FLT: ^18^F-fluorothymidine
^18^F- FDG: ^18^F-fluorodeoxyglucose; HSP90, heat shock protein 90
PI3K: phosphatidylinositol 3'-kinase
mTOR: mammalian target of rapamycin
NF-*κ*B: nuclear factor-kappa B
DNA-PK: DNA-dependent protein kinase
EGFR: epidermal growth factor receptor
ABCG2: ATP-binding cassette subfamily G member 2
P-gp: P-glycoprotein
MRP1: multidrug resistance protein 1
KRAS: V-Ki-ras2 Kirsten rat sarcoma viral oncogene homolog
GLUT1: glucose trasporter-1
HIF1*α*: hypoxia-inducible factor-1 *α*
PET: positron emission tomography
NUV60: normalized uptake value at 60 min post injection
CI: combination index

## Supplementary Material

Supplementary Informations

## References

[bib1] Bose P, Dai Y, Grant S. Histone deacetylase inhibitor (HDACI) mechanisms of action: emerging insights. Pharmacol Ther 2014; 143: 323–336. 24769080 10.1016/j.pharmthera.2014.04.004 PMC4117710

[bib2] Parmigiani RB, Xu WS, Venta-Perez G, Erdjument-Bromage H, Yaneva M, Tempst P et al. HDAC6 is a specific deacetylase of peroxiredoxins and is involved in redox regulation. Proc Natl Acad Sci USA 2008; 105: 9633–9638. 18606987 10.1073/pnas.0803749105 PMC2443817

[bib3] Haggarty SJ, Koeller KM, Wong JC, Grozinger CM, Schreiber SL. Domain-selective small-molecule inhibitor of histone deacetylase 6 (HDAC6)-mediated tubulin deacetylation. Proc Natl Acad Sci USA 2003; 100: 4389–4394. 12677000 10.1073/pnas.0430973100 PMC153564

[bib4] Bali P, Pranpat M, Bradner J, Balasis M, Fiskus W, Guo F et al. Inhibition of histone deacetylase 6 acetylates and disrupts the chaperone function of heat shock protein 90: a novel basis for antileukemia activity of histone deacetylase inhibitors. J Biol Chem 2005; 280: 26729–26734. 15937340 10.1074/jbc.C500186200

[bib5] Witt O, Deubzer HE, Milde T, Oehme I. HDAC family: what are the cancer relevant targets? Cancer Lett 2009; 277: 8–21. 18824292 10.1016/j.canlet.2008.08.016

[bib6] Hubbert C, Guardiola A, Shao R, Kawaguchi Y, Ito A, Nixon A et al. HDAC6 is a microtubule-associated deacetylase. Nature 2002; 417: 455–458. 12024216 10.1038/417455a

[bib7] Santo L, Hideshima T, Kung AL, Tseng JC, Tamang D, Yang M et al. Preclinical activity, pharmacodynamic, and pharmacokinetic properties of a selective HDAC6 inhibitor, ACY-1215, in combination with bortezomib in multiple myeloma. Blood 2012; 119: 2579–2589. 22262760 10.1182/blood-2011-10-387365 PMC3337713

[bib8] Oda K, Stokoe D, Taketani Y, McCormick F. High frequency of coexistent mutations of PIK3CA and PTEN genes in endometrial carcinoma. Cancer Res 2005; 65: 10669–10673. 16322209 10.1158/0008-5472.CAN-05-2620

[bib9] Vivanco I, Sawyers CL. The phosphatidylinositol 3-Kinase AKT pathway in human cancer. Nat Rev Cancer 2002; 2: 489–501. 12094235 10.1038/nrc839

[bib10] Hollander MC, Blumenthal GM, Dennis PA. PTEN loss in the continuum of common cancers, rare syndromes and mouse models. Nat Rev Cancer 2011; 11: 289–301. 21430697 10.1038/nrc3037 PMC6946181

[bib11] Stronach EA, Chen M, Maginn EN, Agarwal R, Mills GB, Wasan H et al. DNA-PK mediates AKT activation and apoptosis inhibition in clinically acquired platinum resistance. Neoplasia 2011; 13: 1069–1080. 22131882 10.1593/neo.111032 PMC3223610

[bib12] Sos ML, Koker M, Weir BA, Heynck S, Rabinovsky R, Zander T et al. PTEN loss contributes to erlotinib resistance in EGFR-mutant lung cancer by activation of Akt and EGFR. Cancer Res 2009; 69: 3256–3261. 19351834 10.1158/0008-5472.CAN-08-4055 PMC2849653

[bib13] Kaliszczak M, Trousil S, Aberg O, Perumal M, Nguyen QD, Aboagye EO. A novel small molecule hydroxamate preferentially inhibits HDAC6 activity and tumour growth. Br J Cancer 2013; 108: 342–350. 23322205 10.1038/bjc.2012.576 PMC3566806

[bib14] Nicholson KM, Anderson NG. The protein kinase B/Akt signalling pathway in human malignancy. Cell Signal 2002; 14: 381–395. 11882383 10.1016/s0898-6568(01)00271-6

[bib15] Butts BD, Kwei KA, Bowden GT, Briehl MM. Elevated basal reactive oxygen species and phospho-Akt in murine keratinocytes resistant to ultraviolet B-induced apoptosis. Mol Carcinog 2003; 37: 149–157. 12884366 10.1002/mc.10131

[bib16] Ocana A, Vera-Badillo F, Al-Mubarak M, Templeton AJ, Corrales-Sanchez V, Diez-Gonzalez L et al. Activation of the PI3K/mTOR/AKT pathway and survival in solid tumors: systematic review and meta-analysis. PloS One 2014; 9: e95219. 24777052 10.1371/journal.pone.0095219 PMC4002433

[bib17] DeGraffenried LA, Fulcher L, Friedrichs WE, Grunwald V, Ray RB, Hidalgo M. Reduced PTEN expression in breast cancer cells confers susceptibility to inhibitors of the PI3 kinase/Akt pathway. Ann Oncol 2004; 15: 1510–1516. 15367412 10.1093/annonc/mdh388

[bib18] Lee C, Kim JS, Waldman T. PTEN gene targeting reveals a radiation-induced size checkpoint in human cancer cells. Cancer Res 2004; 64: 6906–6914. 15466180 10.1158/0008-5472.CAN-04-1767 PMC4384184

[bib19] Vazquez F, Ramaswamy S, Nakamura N, Sellers WR. Phosphorylation of the PTEN tail regulates protein stability and function. Mol Cell Biol 2000; 20: 5010–5018. 10866658 10.1128/mcb.20.14.5010-5018.2000 PMC85951

[bib20] Rahdar M, Inoue T, Meyer T, Zhang J, Vazquez F, Devreotes PN. A phosphorylation-dependent intramolecular interaction regulates the membrane association and activity of the tumor suppressor PTEN. Proc Natl Acad Sci USA 2009; 106: 480–485. 19114656 10.1073/pnas.0811212106 PMC2626728

[bib21] Pore N, Jiang Z, Shu HK, Bernhard E, Kao GD, Maity A. Akt1 activation can augment hypoxia-inducible factor-1alpha expression by increasing protein translation through a mammalian target of rapamycin-independent pathway. Mol Cancer Res 2006; 4: 471–479. 16849522 10.1158/1541-7786.MCR-05-0234

[bib22] Ericson K, Gan C, Cheong I, Rago C, Samuels Y, Velculescu VE et al. Genetic inactivation of AKT1, AKT2, and PDPK1 in human colorectal cancer cells clarifies their roles in tumor growth regulation. Proc Natl Acad Sci USA 2010; 107: 2598–2603. 20133737 10.1073/pnas.0914018107 PMC2823889

[bib23] Leyton J, Alao JP, Da Costa M, Stavropoulou AV, Latigo JR, Perumal M et al. *In vivo* biological activity of the histone deacetylase inhibitor LAQ824 is detectable with 3'-deoxy-3'-[18 F]fluorothymidine positron emission tomography. Cancer Res 2006; 66: 7621–7629. 16885362 10.1158/0008-5472.CAN-05-3962

[bib24] Nakahata S, Ichikawa T, Maneesaay P, Saito Y, Nagai K, Tamura T et al. Loss of NDRG2 expression activates PI3K-AKT signalling via PTEN phosphorylation in ATLL and other cancers. Nat Commun 2014; 5: 3393. 24569712 10.1038/ncomms4393 PMC3948061

[bib25] Song MS, Salmena L, Pandolfi PP. The functions and regulation of the PTEN tumour suppressor. Nat Rev Mol Cell Biol 2012; 13: 283–296. 22473468 10.1038/nrm3330

[bib26] Meng Z, Jia LF, Gan YH. PTEN activation through K163 acetylation by inhibiting HDAC6 contributes to tumour inhibition. Oncogene 2015; 35: 2333–2344. 26279303 10.1038/onc.2015.293

[bib27] Chang JD, Field SJ, Rameh LE, Carpenter CL, Cantley LC. Identification and characterization of a phosphoinositide phosphate kinase homolog. J Biol Chem 2004; 279: 11672–11679. 14701839 10.1074/jbc.M309721200

[bib28] Vichai V, Kirtikara K. Sulforhodamine B colorimetric assay for cytotoxicity screening. Nat Protoc 2006; 1: 1112–1116. 17406391 10.1038/nprot.2006.179

[bib29] Workman P, Aboagye EO, Balkwill F, Balmain A, Bruder G, Chaplin DJ et al. Guidelines for the welfare and use of animals in cancer research. Br J Cancer 2010; 102: 1555–1577. 20502460 10.1038/sj.bjc.6605642 PMC2883160

[bib30] Team RC. R: A language and environment for statistical computing. R Foundation for Statistical Computing, Vienna, Austria. 2013. ISBN 3-900051-07-0; 2014.

[bib31] Benjamini Y, Hochberg Y. Controlling the false discovery rate: a practical and powerful approach to multiple testing. J R Stat Soc B 1995; 57: 289–300.

[bib32] Kaliszczak M, Patel H, Kroll HBS, Carroll L, Smith G, Delaney S et al. Development of a cyclin dependent kinase inhibitor devoid of ABC transporter-dependent drug resistance. Br J Cancer 2013; 109: 2356–2367. 24071597 10.1038/bjc.2013.584 PMC3817326

[bib33] Kaliszczak M, Antonow D, Patel KI, Howard P, Jodrell DI, Thurston DE et al. Optimization of the antitumor activity of sequence-specific pyrrolobenzodiazepine derivatives based on their affinity for ABC transporters. AAPS J 2010; 12: 617–627. 20703960 10.1208/s12248-010-9225-x PMC2977001

