# Supplementary material for: AKT activation controls cell survival in response to HDAC6 inhibition
Source: Cell Death Dis. 2016 Jun 30;7(6):e2286–. doi: 10.1038/cddis.2016.180 (PMC5108334; doi:10.1038/cddis.2016.180)
Supplement: Supplementary Informations [file cddis2016180x1.pdf]

SUPPLEMENTAL DATA

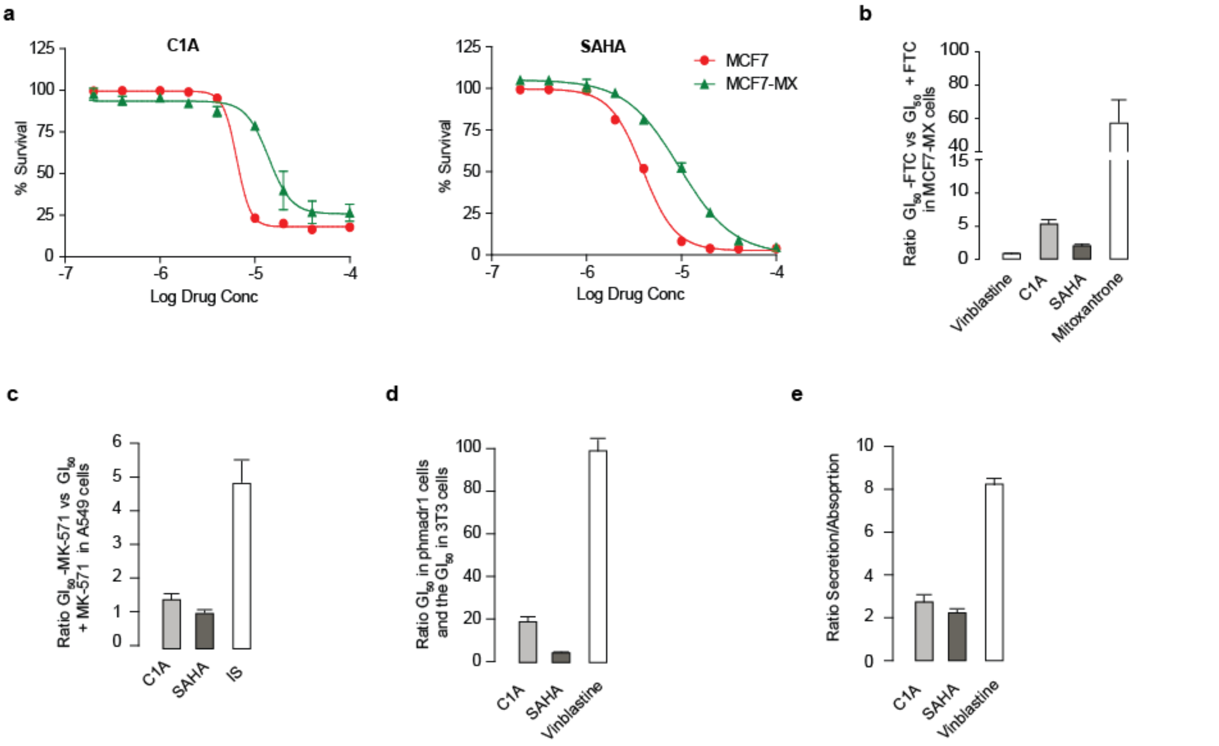

Supplementary Figure S1. C1A is a weak substrate of the ABC transporters, P-gp, ABCG2 and MRP1. (a) Effect of ABCG2 on the cytotoxicity of C1A (left) and SAHA (right) as determined by SRB assay in mitoxantrone resistant cell line, overexpressing ABCG2 (MCF7-MX) and in parental cell line MCF7. (b) Effect of fumitremorgin C (FTC-10 µM), a specific inhibitor of ABCG2, on the cytotoxicity of the compounds in MCF7-MX cells. Results are expressed as a ratio between the growth inhibitory effect (GI50) when cells were untreated with FTC and the GI50 when the cells were treated with the inhibitor. (c) Effect of MK-571 (10 µM), a specific inhibitor of MRP1 on the cytotoxicity of the compounds in A549 cells. (d) Effect of P-gp on the cytotoxicity of the different compounds in pHamdr1 cells, overexpressing the transporter and compared to its isogenic parental cell line 3T3. (e) Effect of the ABC transporters on the permeability of C1A and SAHA compared to the positive control vinblastine as determined by the transwell assay. A compound associated with a ratio between the secretion, i.e. permeability from the basal side to the apical side and the absorption, i.e. permeability from the apical to the basal side greater than 3, is considered as being actively efflux.

Supplementary Table S1. Drug synergy studies. Cytotoxicity was measured at different effective dose (ED) levels of C1A or tubastatin A. Combination index (CI) between C1A and tubastatin A and the different inhibitors of the PI3K/AKT/mTOR signaling pathway (rapamycin, wortmanin, LY-29004, BEZ235, API-2) in HCT-116 cells following 72 h incubation. The different CI have been determined using the effect dose at 50 %, 75 % and 95 % as determined by the SRB assay. Results are mean of n = 3. CI < 1 demonstrates synergism (highlighted in green), CI = 1, additive effect and CI > 1, no significant combination effect. (n.d.; not determined).

	C1A			Tubastatin A		
	ED50	ED75	ED90	ED50	ED75	ED90
Rapamycin (μM)						
1.9	0.82	1.07	1.41			
3.8	0.10	0.17	0.30			
7.5	0.12	0.25	0.51			
15	0.06	0.13	0.29			
Wortmanin (μM)						
1.6	0.33	0.47	0.73	0.33	0.47	0.73
3.1	0.19	0.38	0.81	0.19	0.38	0.81
6.3	0.21	0.42	0.93	0.21	0.42	0.93
12.5	0.07	0.15	0.33	0.07	0.15	0.33
25	0.03	0.09	0.25	0.03	0.09	0.25
LY-29004 (μM)						
0.1	2.1	1.7	1.5			
0.25	1.4	1.3	1.2			
0.5	2.1	1.7	1.5			
1	0.25	0.34	0.50			
2.5	0.32	0.47	0.72			
4	0.36	0.48	0.64			
20	0.03	0.07	0.15			
BEZ-235 (nM)						
0.16	*n.d.*	*n.d.*	*n.d.*	1.86	1.30	1.08
0.8	*n.d.*	*n.d.*	*n.d.*	0.24	0.31	0.45
4	0.36	0.43	0.54	0.17	0.23	0.39
20	0.08	0.14	0.25	0.02	0.05	0.10
100	0.02	0.05	0.11	0.02	0.03	0.09
API-2 (μM)						
0.1	0.52	0.59	0.73			
0.25	0.49	0.55	0.68			
1	0.23	0.32	0.48			
2.5	0.29	0.43	0.69			
10	0.27	0.38	0.59			
25	0.10	0.16	0.28			
